# Optimization of a Cryopreservation Method for the Endangered Korean Species *Pogostemon yatabeanus* Using a Systematic Approach: The Key Role of Ammonium and Growth Regulators

**DOI:** 10.3390/plants10102018

**Published:** 2021-09-26

**Authors:** Hyo-Eun Lee, Elena Popova, Ha-Na Park, Sang-Un Park, Haeng-Hoon Kim

**Affiliations:** 1 Department of Agricultural Life Science, Sunchon National University, Suncheon 57922, Korea; yee0430@scnu.ac.kr (H.-E.L.); qkrgksk1102@naver.com (H.-N.P.); 2 K.A. Timiryazev Institute of Plant Physiology of Russian Academy of Sciences, 127276 Moscow, Russia; elena_aygol@hotmail.com; 3 Division of Plant Science and Resources, Chungnam National University, Daejeon 34134, Korea; supark@cnu.ac.kr

**Keywords:** alternative vitrification solutions, ammonium stress, droplet-vitrification, endangered species conservation, osmoprotection, plant growth regulators, regrowth medium

## Abstract

Cryopreservation provides a secure long-term conservation option for rare and endangered plant species with non-orthodox or limitedly available seeds. Wide application of cryopreservation to biobank wild flora is hampered by the need to re-optimize nearly all protocol steps for every new species. We applied a systematic approach to simplify optimization of a multi-stage droplet-vitrification method for the endangered wetland Korean species, *Pogostemon yatabeanus*. This approach consisted of a standard procedure pre-selected based on material type and size, which was complemented with 11 additional treatments to reveal the most impactful conditions. Effect of ammonium nitrate at various protocol steps was also tested. The highest shoot tip survival (92%) and plant regeneration (90%) after cryopreservation were achieved using preculture with 10% sucrose followed by 40 min osmoprotection and 60 min treatment with vitrification solution A3-80% (33.3% glycerol + 13.3% dimethyl sulfoxide + 13.3% ethylene glycol + 20.1% sucrose) on ice. A three-step regrowth procedure starting with ammonium-free medium with 1 mg/L GA_3_ and 1 mg/L BA followed by ammonium-containing medium with and without growth regulators was essential for the development of healthy plants from cryopreserved shoot tips. This approach enables fast optimization of the cryopreservation procedure for new osmotic stress-sensitive plant species.

## 1. Introduction

Target eight of the Global Strategy for Plant Conservation (GSPC) had an ambitious goal to save 75% of threatened plant species in ex situ collections and make at least 20% of them available for recovery and restoration programs [1]. However, the 2020 GSPC progress report revealed that over half of endemic threatened species were not conserved ex situ within their countries of origin, implying reduced availability of plant material for ecological or species restoration [2]. Major difficulties have been observed with “exceptional species” producing seeds that do not tolerate conventional seed bank conditions, seeds that are dormant or low in number [2,3]. Not surprisingly, many of such species originate from the tropics and/or wetlands and do not possess inherited desiccation and chilling tolerance [4]. 

Cryopreservation combined with in vitro technologies offers a new valuable option for long-term conservation and restoration of such exceptional species [5,6,7]. Cryopreservation, i.e., storage of viable material in liquid nitrogen (LN, −196 °C), ensures long-term gene pool conservation while in vitro plant multiplication provides a sufficient number of healthy plants to support species restoration or relocation to new habitats [8,9,10,11,12]. 

Among modern methods of plant cryopreservation, droplet-vitrification (D-V) is one of the most popular and widely applicable techniques [13]. It has been utilized for large-scale cryopreservation of clonal crop collections, e.g., to *Musa* spp. collection at the Bioversity International Transit Center (ITC, Belgium, [14]), *Allium* spp. collection at Rural Development Administration (RDA, Rep. Korea, [15]), potato collections at the International Potato Center (CIP, Peru, [16]) and the N.I. Vavilov All-Russian Institute of Plant Genetic Resources [17]. Modifications of this method have been effective for cryobanking fruit trees, woody shrubs, ornamental and aromatic plants of high economic value [18,19,20]. There is also a growing number of successful examples of D-V application for cryobanking wild flora [6]; however, these cases are few compared to clonal crops. There are several reasons why wider application of D-V to conserve endangered plant species is hampered worldwide. These include limited amount of starting material available for research, unknown desiccation and chilling tolerance of new species, and non-optimized (and often not even existing) protocols for their in vitro multiplication. However, the greatest limitations are, perhaps, the absence of the uniform cryopreservation protocol applicable to all plants, and the large spectrum of physiological responses to cryopreservation stress observed among species [4]. 

D-V is a complex procedure, which involves several steps, namely preculture, osmoprotection, vitrification solution (VS) treatment, rewarming, unloading (washing off the cryoprotectants), and regrowth. Each step may become critical for post-cryopreservation survival and regeneration of plant material [21,22]. So far, there is no universal rule or clear guidelines leading to successful implementation of this method for most plant species. Often the conditions at each step should be optimized de novo for every new species to be conserved [23], which require dozens of explants. However, it is not uncommon, that significant variation in survival during the cryopreservation procedure is observed among different cultivars within the same species, which implies that a response of plant material to cryopreservation stress may be genotype-dependent [17,18,20,24]. The need for “re-optimization” of the already established procedure further complicates the development of the effective cryopreservation methods for endangered plant species with limited source material available for the experiments [7]. 

To overcome this shortage, a systematic approach to protocol development has been formulated and successfully tested on a range of wild and cultivated plants [25,26]. In this approach, cryopreservation starts with a standard procedure, which is further adopted to a particular species in a set of predetermined treatments involving alternative osmoprotection and vitrification solutions [27,28]. This approach enabled successful cryopreservation of the number of endangered species, including *Betula lenta* L. [3], *Castilleja levisecta* Greenm. [10], *Lupinus rivularis* Lindl. [29], *Aster altaicus* Willd. var. *uchiyamae* [30], and *Dendrobium moniliforme* (L.) Sw. [31]. In the present study, we explore the feasibility of using this approach to cryopreserve *Pogostemon yatabeanus*, an endangered wetland Korean species [32], using shoot tips of in vitro propagated plants. *P. yatabeanus* usually occurs in swamps and has strongly dormant seeds with a low (ca. 3%) germination rate [33]. The species is threatened by over-exploration, habitat loss and climate change and was recently included in the Korean Red List [32]. The initial, or standard, cryopreservation procedure for the species was developed based on the preliminary study [34] and complemented with a preset of preculture and cryoprotectant treatments aimed to evaluate shoot tip response to osmotic and chemical stresses. 

The majority of the cryopreservation studies for the new species are focused solely on the optimization of the pre-LN protocol steps, i.e., preculture and cryoprotectant treatments. Several researchers, e.g., [3,35], also considered the effect of unloading (removing of cryoprotectants). Regretfully, few studies have explored the effect of post-cryopreservation (regrowth) conditions (light, medium composition and growth regulators), however, at least for some species, these factors may have a major impact on regeneration of healthy plantlets from cryopreserved materials [18,36,37,38]. Our preliminary study with *P. yatabeanus* revealed that, despite a relatively high (>80%) survival of cryopreserved shoot tips, their development into the whole plants required ammonium-free medium during the first five days after rewarming [34]. Moreover, step-wise transfer of regenerating shoot tips to the medium with and without growth regulators was also important. Several authors [30,39,40,41,42,43,44] have also highlighted the benefit of using ammonium-free medium at various protocol steps and the need to optimize growth regulators for better plant regeneration after cryopreservation; however, these studies are scarce. The aim of the present study was to investigate the effect of ammonium ion at various steps of the D-V protocol and to improve regeneration of healthy plants from cryopreserved shoot tips by testing various combinations of regrowth conditions and growth regulator content in the regrowth medium.

## 2. Results

### 2.1. Cryoprotection and Regrowth of Pogostemon yatabeanus Shoot Tips: Effect of Ammonium

#### 2.1.1. Effect of Ammonium at Different Protocol Stages (Experiment 1)

Omission of ammonium nitrate from the nutrient medium was tested during the course of the droplet-vitrification procedure, including preculture, osmoprotection, cryoprotection, unloading and regrowth (Table 1). 

There was no significant difference (*p* = 0.0792) in survival of cryoprotected shoot tips among the treatments tested, while regeneration was significantly (*p* < 0.0001) affected by the presence of ammonium at various protocol stages. The highest (95.7%) plant regeneration was recorded when regrowth was performed on ammonium-free medium for the initial 5 days (Table 1, treatment 4). Regrowth on ammonium-free medium for 1 day (treatment 6) or 3 days (treatment 5) proved to be less beneficial, although the differences from the best treatment were insignificant. Initial regrowth on ammonium-containing medium (treatment 7) produced the lowest regeneration. 

Removing of ammonium from the medium used at preculture, osmoprotection, cryoprotection and unloading steps resulted in significantly (*p* < 0.0001) lower regeneration (70.1–82.6%) regardless of the ammonium presence in regrowth medium (treatments 1–3). These results suggested that regrowth on ammonium-free medium for the initial 5 days after D-V treatment was the best option for normal plant regeneration from cryoprotected shoot tips. 

#### 2.1.2. Substitution of Ammonium and Modification of Regrowth Conditions (Experiment 2)

In Experiment 2, ammonium nitrate in the medium used for preparing cryoprotectant solution and for regrowth was fully or partially substituted by NaNO_3_. Addition of activated charcoal in the medium for regrowth and light exposure during the first regrowth steps were also tested. As a result, there was no significant difference in survival of cryoprotected shoot tips among the treatments (Table 2, *p* = 0.1464). By contrast, plant regeneration from shoot tips was significantly (*p* < 0.0001) affected by nitrogen source. High regeneration (87.7–96.2%) was obtained when ammonium-free RM1 medium was used at first regrowth step (treatments 2, 3 and 8) followed by treatments where ammonium was fully or partially substituted by sodium nitrate (80.1–87.7%, treatments 4–6). The lowest regeneration of 70.0–75.6% was observed with initial regrowth on ammonium-containing medium RM2 (treatments 1, 7, 9). The highest regeneration (94.6–96.2%) was recorded when initial regrowth was performed on ammonium-free medium for 5 days and was not affected by a frequent (weekly) transfer of shoot tips to a fresh medium during the next steps (treatments 2 and 3).

Including activated charcoal into regrowth medium (treatments 7 and 8) showed no significant effect on regeneration percentage compared to treatments without AC (treatments 1 and 2). Regrowth under light (treatments 9 and 10) reduced regeneration on ammonium-free RM1 medium by 10% but had no effect on regeneration on ammonium-containing RM2 medium. 

As a result of this experiment, ammonium substitution in the regrowth medium was found to be ineffective compared to the regrowth on ammonium-free medium for at least 5 days after treatment.

#### 2.1.3. Impact of Vitrification Solution Treatment (Experiment 3)

**Figure 1:**
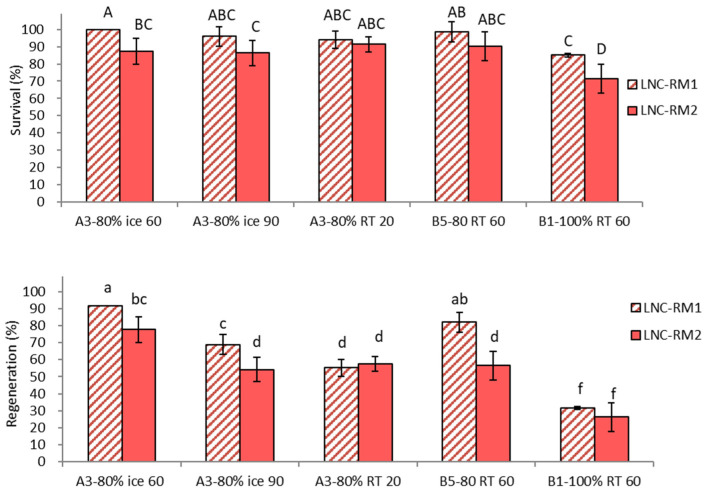
Effect of vitrification solution treatment conditions and initial (first 5 days) regrowth on ammonium-free (RM1) or ammonium–containing (RM2) medium on survival and regeneration of *Pogostemon yatabeanus* shoot tips in the droplet-vitrification procedure. Shoot tips were cryoprotected but not cryopreserved. Treatment conditions are listed in the table in Section 4.2. (LNC).

Cryoprotection treatments, including diverse options of vitrification solution, duration and temperature tested, significantly (*p* < 0.0001) affected survival and regeneration of shoot tips (Figure 1). Regeneration of shoot tips with non-optimum cryoprotection, e.g., following treatments with highly concentrated vitrification solution PVS3 (B1-100% RT 60, 26.2%), at room temperature (A3-80% RT 20, 57.4%), and for a long time period (A3-80% ice 90, 54.2%) was significantly lower compared to the standard procedure (A3-80% ice 60, 91.4%) irrespective of ammonium ion presence in regrowth medium. 

Ammonium presence in regrowth medium also had a significant (*p* < 0.0001) impact on both survival and regeneration of cryoprotected shoot tips (Figure 1). In three of five cryoprotection treatments, initial regrowth on ammonium-free medium RM1 produced ca. 25% higher regeneration (71.9–91.4%) compared to regrowth on ammonium-containing medium RM2 (54.2–77.6%). Treatments with A3-80% at room temperature for 20 min (A3-80% RT 20) and PVS3 at room temperature for 60 min (B1-100% RT 60) resulted in similarly low regrowth on both RM1 and RM2 medium, which indicated that shoot tips in these treatments were severely affected by cytotoxicity of vitrification solutions and were unable to recover even on ammonium-free medium. 

As a result of this experiment, the best cryoprotection options for shoot tips, A3-80% on ice for 60 min and B5-80% at room temperature for 60 min, have been selected for further experiments, including LN exposure. Treatment with A3-80% on ice for 90 min was also included to test the potent beneficial effect of long cryoprotection on the recovery after cryopreservation.

#### 2.1.4. Impact of Individual Protocol Steps (Experiment 3, Continued)

The impact of individual steps of the cryopreservation protocol (preculture, osmoprotection and vitrification solution treatment) were tested in combination of ammonium-free (RM1) and ammonium-containing (RM2) medium at regrowth step 1 using the best VS treatment (A3-80% ice 60 min) revealed during the previous experiment. 

**Figure 2:**
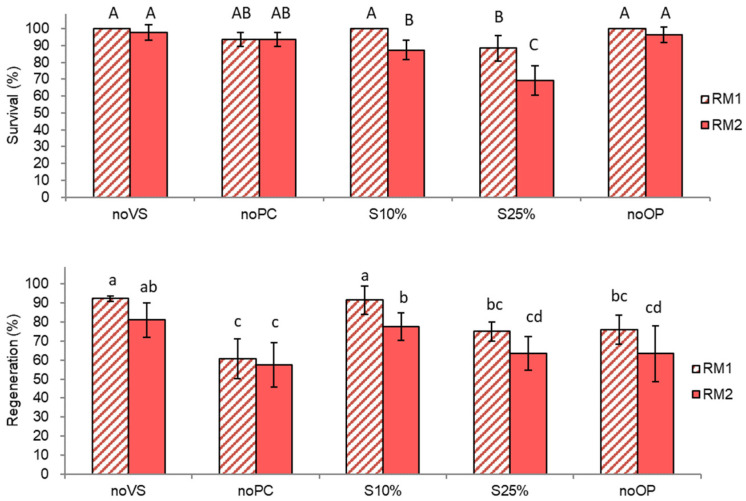
Effect of individual pre-LN protocol steps (preculture, osmoprotection, cryoprotection) and initial (first 5 days) regrowth on ammonium-free (RM1) or ammonium–containing (RM2) medium on survival and regeneration of *Pogostemon yatabeanus* shoot tips in the droplet-vitrification procedure. Shoot tips were cryoprotected but not cryopreserved. Treatment conditions are listed in the table in Section 4.2. (LNC).

Both survival and regeneration of shoot tips were significantly (*p* < 0.0001) affected by the protocol step modification as well as by ammonium presence in regrowth medium (Figure 2). 

Highest survival (100%) and regeneration (92%) were observed in treatments, including preculture with 10% sucrose for 31 h, osmoprotection and regrowth on ammonium-free medium regardless of vitrification solution exposure (S10%-RM1 and noVS-RM1) indicating that these treatments were optimum to enable normal shoot tip development after the cryoprotection procedure. Step-wise preculture in 10–25% sucrose (S25%-RM1 and S25%-RM2) significantly reduced both survival and regeneration of cryoprotected shoot tips. Skipping the osmoprotection treatment decreased regeneration compared to the standard protocol by 17% when regrowth was performed on ammonium-free medium (noOP-RM1) and by 28% following regrowth on ammonium-containing medium (noOP-RM2). The lowest regeneration (58–61%) was observed in the treatment without preculture regardless of the ammonium presence in regrowth medium (noPC-RM1 and noPC-RM2) indicating that not-precultured shoot tips were unable to overcome the severe osmotic shock caused by highly concentrated cryoprotectant solutions even when recovered on ammonium-free medium. 

With all the conditions tested, regeneration decreased if shoot tips were recovered for the initial 5 days on ammonium-containing medium (58–81%) compared to shoot tips recovered on ammonium-free medium (74–92%), except for the treatment without preculture (noPC). Even in the treatment without vitrification solution (which is usually the most injurious cryoprotection step), shoot tips on ammonium-containing medium showed similar regeneration (81.0%) as after the standard cryoprotection with VS A3-80% on ice for 60 min (77.6%) (treatments “noVS-RM2” vs. “S-10%-RM2”). It is likely that the stress induced during the osmoprotection with C4-35% for 40 min was further accelerated by regrowth on ammonium-containing medium (RM2).

### 2.2. Cryopreservation of Pogostemon yatabeanus Shoot Tips 

#### 2.2.1. Impact of Individual Protocol Steps and Vitrification Solution Treatment after Cryopreservation (Experiment 3, Continued)

At this stage, best options for cryoprotection selected in the previous experiments were used to cryopreserve *P. yatabeanus* shoot tips. In addition, effect of individual protocol steps on survival and regeneration of LN-exposed shoot tips were investigated (Table in Section 4.2., LN). Regrowth was always performed on ammonium-free RM1 medium for the first 5 days followed by ammonium-containing medium as described in the standard protocol.

**Figure 3:**
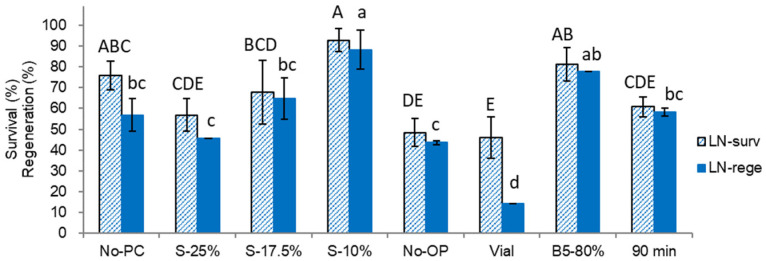
Effect of preculture, osmoprotection, cryoprotection and container for cooling/rewarming on survival (surv) and regeneration (rege) of cryopreserved *Pogostemon* *yatabeanus* shoot tips in the droplet-vitrification procedure. Treatment S-10% represents a standard protocol: preculture with 10% sucrose for 31 h, osmoprotection with C4-35% for 40 min, and cryoprotection with A3-80% on ice for 60 min, followed by cooling and warming using aluminum foil strips. Treatments are listed in the table in Section 4.2. (LN).

Various steps of the cryopreservation protocol significantly affected both survival (*p* = 0.0004) and regeneration (*p* = 0.0016) of cryopreserved shoot tips. As in the previous experiments, shoot tips were found sensitive to osmotic shock as evidenced by their poor response to treatment without preculture (no-PC) and high sucrose concentration (17.5–25%) in preculture medium (Figure 3). A progressive preculture with 10% sucrose followed by 25% sucrose (S-25%) produced the lowest regeneration (45.5%) of cryopreserved shoot tips. The highest regeneration of cryopreserved shoot tips (88%) was gained after preculture with 10% sucrose (S-10%). In all preculture treatments tested, there was no significant difference between regeneration of cryoprotected (Figure 2) and cryopreserved (Figure 3) shoot tips, which indicated that the damages causing low regeneration in other treatments (S-25%, no-PC, S-17.5%) were mostly attributed to a pre-LN stage. 

Even precultured shoot tips were sensitive to osmotic stress induced by direct exposure to vitrification solution A3-80% without osmoprotection (Figure 3, no-OP) with survival and regeneration cryopreserved shoot tips decreasing to 30–48%. 

Cooling and rewarming using 2-mL cryovials produced 46% survival and 14% regeneration after cryopreservation (Figure 3, Vial), indicating that shoot tips were not sufficiently cryoprotected and desiccated via treatment with A3-80% and were likely damaged by intracellular ice crystallization and recrystallization during cooling and rewarming procedure in vials. In such a case, higher cooling and warming rates provided by aluminum foil strips in a droplet-vitrification method were critical for the successful regeneration of cryopreserved shoot tips.

Alternative cryoprotection treatments with B5-80% for 60 min (Figure 3, B5-80%) and A3-80% for 90 min (Figure 3, 90 min) produced slightly lower post-cryopreservation regeneration compared to the standard cryoprotection with A3-80% for 60 min (Figure 3 S-10%).

#### 2.2.2. Effect of Growth Regulators in Regrowth Medium (Experiment 4)

In the present study, we investigated combinational effects of plant growth regulators during steps 1 and 2 of the three-step regrowth procedure: step 1, ammonium-free medium with growth regulators; step 2, full-strength medium with growth regulators; and step 3, full-strength medium without growth regulators.

**Figure 4:**
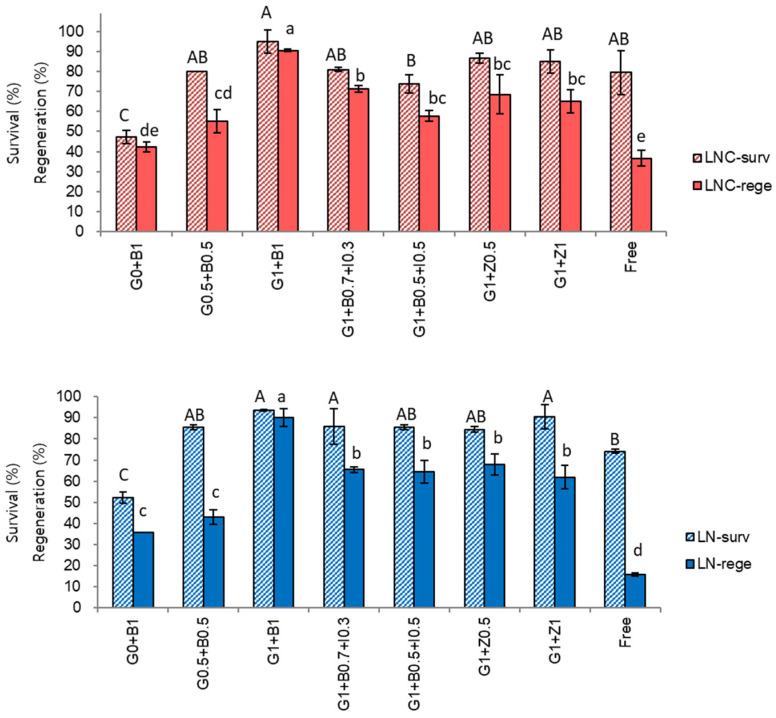
Effect of growth regulators in regrowth medium (steps 1 and 2 of the three-step regrowth procedure) on survival (surv) and regeneration (rege) of cryoprotected (LNC) and cryopreserved (LN) *Pogostemon yatabeanus* shoot tips in the droplet-vitrification procedure. G0, G1-0 and 1 mg/L gibberellic acid-3; B0, B0.5, B0.7 and B1-0, 0.5, 0.7 and 1 mg/L benzyl adenine; Z1-1 mg/L zeatin; I0.3 and I0.5-0.3 and 0.5 mg/L indolyl acetic acid; Free—medium without growth regulators.

The composition of growth regulators in regrowth medium significantly (*p* < 0.0001) affected survival and regeneration of cryoprotected and cryopreserved shoot tips (Figure 4). The highest survival (95%) and regeneration (90%) of both cryoprotected and cryopreserved shoot tips were recorded on regrowth medium with 1 mg/L gibberellic acid (GA_3_) and 1 mg/L benzyl adenine (BA) during the initial two steps of recovery (Figure 4, G1 + B1, standard treatment). This is 20% higher than regeneration achieved from the best treatment in our previous study [34]. 

The omission of GA_3_ (G0 + B1) resulted in reduced survival (52.3%) and regeneration (35.7%) of both cryoprotected and cryopreserved shoot tips, demonstrating that GA_3_ was important at both stages of the regrowth. Low concentration of GA_3_ and BA (G0.5 + B0.5) also caused decreasing in the survival (80.0–85.6%) and regeneration (43.1–55.0%) of both cryoprotected and cryopreserved explants. Other combinations of growth regulators tested, including IAA with low concentrations of BA and GA_3_ (G1 + B0.7 + I0.3, G1 + B0.5 + I0.5) and substitution of BA with zeatin (G1 + Z0.5, G1 + Z1), were also less effective compared to the standard treatment producing relatively low survival (62–86%) and regeneration (58–71%) of cryoprotected and cryopreserved shoot tips. The lowest survival (74.2–79.4%) and regeneration (16.0–36.7%) of both LNC and LN was recorded on growth regulator-free medium. 

These results suggest that cryopreserved shoot tips of *P. yatabeanus* require a step-wise regrowth on medium with GA_3_ and BA to maximize regeneration of plantlets. With optimized pre-LN and post-LN treatments, *P. yatabeanus* shoot tips produced 93.5–95.0% survival and 90.2–90.5% regeneration after both cryoprotection and cryopreservation even though this material is sensitive to the osmotic stress and the toxicity of highly concentrated vitrification solutions.

#### 2.2.3. Combination of Growth Regulators and Ammonium-Free Regrowth Medium at Three Regrowth Steps (Experiment 5)

In this experiment, we investigated combinational effects of ammonium-free medium and plant growth regulators at regrowth steps 1, 2 and 3 (Table 3, Figure S1). The highest survival and regeneration of both cryoprotected (LNC) and cryopreserved (LN) shoot tips were obtained with standard conditions employing step 1 on ammonium-free medium with 1 mg/L GA_3_ and 1 mg/L BA followed by step 2 using ammonium-containing medium with the same growth regulators and step 3 on ammonium-containing medium without growth regulators (treatment 1). The second-best option (treatment 7) contained the same treatments with the only difference of including growth regulators at step 3. Combination of ammonium-containing medium at step 1 with the absence of growth regulators (treatment 6) was the most harmful and produced only 7.9% regeneration after cryopreservation. 

Using growth regulator-free medium at all three steps (treatments 4 and 6) significantly reduced regeneration of cryopreserved (LN) shoot tips compared to shoot tips treated in the same protocol without cryopreservation (LNC), indicating that the presence of growth regulators was critical for normal regeneration after LN exposure. Omission of growth regulators at step 2 (treatments 2, 4 and 6) was always detrimental and produced low percentage of regeneration in cryopreserved shoot tips regardless of ammonium and growth regulators at step 1. Moreover, including GA_3_ and BA at step 2, was even more impactful than at step 1 (treatment 3 vs. treatment 2). 

Overall, survival and regeneration of shoot tips after cryopreservation were more seriously affected by regrowth conditions compared to cryoprotected shoot tips demonstrating that the nature of the toxic effect of ammonium-ion in step 1 and beneficial effect of growth hormones in step 2 are relative to the stress experienced during the course of the vitrification procedure. 

In conclusion, the most impactful conditions for normal regeneration of LNC and LN *P. yatabeanus* shoot tips were: growth regulators at step 2 > ammonium-free medium at step 1 > growth regulators at step 1 > growth regulator-free medium at step 3.

## 3. Discussion

As a solution-based vitrification method, droplet-vitrification is a multi-step procedure with a number of impactful factors starting from material selection and preparation (mother plant, explant) to pre-LN treatments (preculture, osmoprotection, cryoprotection), cooling in LN and rewarming (container) followed by cryoprotectant removing (unloading) to post-LN steps (regrowth). In our previous study on *Pogostemon yatabeanus* using droplet-vitrification procedure, regeneration of plants from cryopreserved shoot tips remained below 20%, with no improvement after modifications of pre-LN stages (preculture, cryoprotection) [34]. Due to non-optimum regrowth conditions used in the experiments, the impact of VS treatments as well as individual protocol steps remained unknown as all the treatments tested produced similarly low regrowth [34]. Significant improvement in plant regeneration (up to 73%) was achieved only after switching to a three-step regrowth procedure starting with ammonium-free medium followed by medium containing growth regulators (GA_3_ and BA) and then by medium without growth regulators [34]. However, it remained unclear which factor (ammonium-free medium, growth regulators or a sequential transfer to a fresh medium) was the key to the success. Therefore, in this study, we further explored the effects of pre-LN conditions and ammonium ion alone or in combinations with growth regulators in regrowth medium on recovery of cryoprotected and cryopreserved shoot tips. 

In addition to intracellular ice crystallization, extensive cellular dehydration [45], osmotic stress and chemical toxicity [46], oxidative stress induced by reactive oxygen species (ROS) [47,48] is thought to have a negative impact on recovery of cryopreserved samples. Earlier studies indicated that the presence of ammonium may be harmful for plant materials experiencing severe stress [39]. In plants, ammonium toxicity has been related, among other effects, with deregulation of pH homeostasis, ion and hormonal imbalance, impaired nitrate signaling, and oxidative stress [49,50]. In cryopreservation, detrimental effect of ammonium was concentration-dependent and even low concentration of NH_4_NO_3_ negatively affected survival and regeneration of cryopreserved plant cells and shoot tips [37,41,51,52]. Significant improvement of regeneration of cryopreserved shoot tips on ammonium-free compared to ammonium-containing medium was reported for *Holostemma annulare* (Roxb.) K. Schum. (by 26–36% [41]), *Ipomoea batatas* (L.) Lam (by 61% [43], by 46% [44]), *Chrysanthemum morifolium* Ramat. *var. ‘Borami’* (by 36–38% [53]), *Citrus limon* (L.) Osbeck *var. ‘Frost Eureca limon’* (by 17% [54]), and *Aster altaicus* Willd. (by 33% [30]). Avoiding ammonium during the initial 1, 3 or 7 days after cryopreservation was equally beneficial for the viability of cryopreserved *Lavandula vera* DC. and *Oryza sativa* L. cells [51,52]. Substitution of ammonium nitrate by KNO_3_ during cold hardening, cryoprotection, unloading and regrowth increased the recovery of *Betula pendula* Roth shoot tips cryopreserved using slow-freezing method by 53–58% compared to ammonium-containing medium [39]. Similarly, application of ammonium-free medium at preconditioning, preculture and regrowth steps improved regeneration of cryopreserved *Holostemma annulare* shoot tips by 43% compared to the same treatments performed with ammonium-containing medium [41]. Alternatively, Jitsopakul et al. [55] reported higher survival and development of cryopreserved orchid protocorms on ammonium-containing regrowth medium compared with ammonium-free medium (66% vs. 32%) in a droplet-vitrification method, while no effect of ammonium was recorded during regrowth of cryopreserved shoot tips of *Dioscorea alata* L. [37] which possibly implies that, in line with ammonium toxicity in plants [50], the toxic effect of ammonium during cryopreservation is material and genus-specific. 

In our study, 1–3 days regrowth on ammonium-free medium was less effective compared to 5 days, most likely due to the larger size of shoot tips compared to cells. Moreover, exclusion of ammonium from the medium for cryoprotection and regrowth was more beneficial than its substitution or co-presence with NaNO_3_ despite the fact that nitrate (NO_3_^−^) is known to alleviate some toxic effects of NH4^+^ [56]. It is also interesting that ammonium was only harmful during the initial regrowth stage (5 days) and its exclusion from the cryoprotectant solutions (pre-LN steps) or omission during the whole regrowth procedure reduced regeneration of cryoprotected *P. yatabeanus* shoot tips. 

A harmful effect of ammonium-containing medium was triggered by inappropriate cryoprotection procedure such as high sucrose concentration (25%) during preculture, no-osmoprotection, and longer duration of VS treatment (treatments A3-80% ice 90 min, B5-80% RT 60 min). At the same time, severely injured shoot tips were unable to recover even on ammonium-free regrowth medium (treatments without preculture or with highly concentrated VS B1-100% RT 60 min). In line with our study, initial regrowth on ammonium-free medium significantly improved recovery of cryoprotected (66 vs. 98%) and cryopreserved (32 vs. 93%) sweet-potato shoot tips treated with vitrification solution PVS2, which was proven to be highly toxic to plant materials [43]. 

Ammonium is a vital nitrogen source for plants, however, its usage during cryopreservation, particularly under non-optimized cryoprotection conditions, requires careful consideration. The mechanisms by which ammonium inhibits regeneration of plants from cryopreserved shoot tips is not yet clear. Based on the literature, it may be speculated that ammonium in the regrowth medium aggravates the oxidative stress, which occurs in plant tissues during the cryopreservation process. There is evidence that ROS are formed at all stages of the cryopreservation protocol. Le et al. [57] found that oxidative stress was maximized during vitrification solution treatment, LN exposure and unloading. In the present study, shoot tips that were precultured and osmoprotected (Figure 2, noVS-RM2) showed similar regeneration compared to shoot tips treated with vitrification solution (Figure 2, 10%-RM2) when regrown on ammonium-containing medium, hence the oxidative stress could already develop during the osmoprotection step and was likely escalated by ammonium presence in the medium. Cryopreservation stress may reduce metabolic activity of the explants, and the key enzymes of ammonia nitrogen metabolism could be inactivated or retarded several days after rewarming, leading to the accumulation of the toxic levels of ammonium [40]. Hence, omission of ammonium in the regrowth medium for the first 5–7 days may be beneficial for normal explant development and plant regeneration. 

Plant growth regulators (hormones) play a key role in the regeneration of cryopreserved plant materials [36,37,38]. Similar to our study, GA_3_ alone or in combination with BA or other cytokinins usually produces good results promoting direct plant formation from cryopreserved shoot tips [36]. However, regrowth after cryopreservation usually happens in one stage. In the case of *P. yatabeanus* shoot tips, a three-step sequential regrowth treatment was necessary for the development of normal plants, and the presence or absence of growth regulators at each stage was vitally important. The best procedure included initial regrowth on ammonium-free medium with GA_3_ and BA (step 1) followed by normal medium with GA_3_ and BA (step 2) and then normal medium without growth regulators (step 3). With the optimized pre-LN procedure, shoot tips produced 94.9% survival and 91.6% regeneration after cryopreservation even though this material was sensitive to the osmotic stress and to the cytotoxicity by highly concentrated vitrification solutions. Similarly, step-wise regrowth starting with ammonium-free medium and the presence of growth regulators were beneficial for normal plant regeneration from cryopreserved shoot tips of *Ipomoea batatas* and *Chrysanthemum morifolium* var. ‘Yes Morning” that were also osmotic-sensitive [44,53]. Cryopreserved shoot tips of *Dioscorea alata* produced a maximum of 39% shoot regeneration when initially cultured for 40 days on MS medium M2 containing 1/5 NH_4_NO_3_ and a combination of 1.0 mg/L BA, 1.0 mg/L zeatin, 0.15 mg/L IAA and 0.2 mg/L GA_3_ followed by two sequential transfers to other media to promote normal shoot formation [37]. The hypothesis that plant materials that are sensitive to osmotic and chemical toxicity of cryoprotectants may benefit from the step-wise regrowth starting with ammonium-free medium with GA_3_ and BA followed by transfer to ammonium-containing medium with and without growth regulators requires further investigation.

## 4. Materials and Methods

### 4.1. Plant Material

In vitro plants of *Pogostemon yatabeanus* (Makino) Press [58] (synonym *Dysophylla yatabeana* Makino) were grown from seeds collected in nature and germinated in vitro following hot water treatment and three months of cold stratification as described previously [34]. Plants were multiplied on MS medium [59] with 30 g/L sucrose and 2.3 g/L gelrite in 300 mL Gaooze^TM^ culture vessels. Cultures were kept at 25 ± 1 °C under a 16/8 h light/dark photoperiod and 40 µE/(m^2^ s) light intensity. Shoot tips, 1.5 mm long with 1–2 lateral leaves, were extracted from 4–5-day-old single node cuttings and used for cryopreservation. 

### 4.2. Shoot Tip Cryopreservation by Droplet-Vitrification 

The systematic approach used in this study was based on a standard protocol combined with additional treatments designed as single-factor modifications of standard conditions to reveal the response of plant material to osmotic and chemical stress at various stages of the cryopreservation procedure, and identify the most critical factors/steps, if any. The standard procedure was selected from the list of standard protocols developed and published earlier [25,26] based on material type and size. 

*Standard procedure* (indicated as “standard” in Table 4). Shoot tips immediately after excision were precultured in a liquid MS medium with 10% sucrose (S-10%) for 31 h, osmoprotected with a solution C4-35% (17.5% glycerol + 17.5% sucrose) for 40 min, then cryoprotected with vitrification solution A3-80% (33.3% glycerol + 13.3% dimethyl sulfoxide + 13.3% ethylene glycol + 20.1% sucrose) for 60 min on ice using a thermos block. Cryoprotected shoot tips were placed in 5 µl droplets of ice-cold A3-80% on aluminum foil strips (7 × 20 mm), that were plunged directly in LN for minimum 1 h. For rewarming, foil strips with shoot tips were transferred to 20 mL pre-heated (40 °C) 35% sucrose (S-35%) solution and kept for 40 min, with the sucrose solution been replaced after the first 15 min. The explants retrieved from S-35% were blotted dry on filter paper and transferred to regrowth medium 1 (RM1) composed of NH_4_NO_3_–free MS medium with 1 mg/L gibberellic acid (GA_3_), 1 mg/L benzyl adenine (BA), 30 g/L sucrose, 2.2 g/L gelrite, and cultured in the dark for 5 days. Next, explants were transferred to RM2 medium composed of full-strength MS medium with 1 mg/L GA_3_, 1 mg/L BA, 30 g/L sucrose, 2.2 g/L gelrite and cultured under the indirect illumination (dim light) provided by one fluorescent lamp, 40 µE/(m^2^ s), for 23 days. The developed shoots were further transferred to MSF medium, composed of hormone-free MS medium with 30 g/L sucrose and 2.2 g/L gelrite under the same illumination, 40 µE/(m^2^ s), for 2 weeks.

Optimization of the cryopreservation protocol and conditions for regrowth were performed in sequential experiments. During optimization of each individual factor, other conditions remained the same as in the standard protocol. Regrowth of cryoprotected and cryopreserved shoot tips was always performed in three steps. Cryoprotected shoot tips (LNC) were treated in the same way, except they were not exposed to LN.

*Experiment 1. Effect of ammonium at different protocol stages.* In this experiment, ammonium ion was selectively excluded from the medium used for preparing cryoprotectant solutions that were applied at various protocol stages as well as from media for regrowth (Table 1). Shoot tips in this experiment were cryoprotected and washed in S-35% solution but not cryopreserved (LNC only).

*Experiment 2. Substitution of ammonium ion and modification of regrowth conditions.* In this experiment, ammonium nitrate omission or substitution by NaNO_3_ in cryoprotectant and/or regrowth media was tested in combination with various regrowth conditions, including light, activated charcoal and frequent transfer to a fresh medium (Table 2). Commercial Duchefa mixtures were used to prepare the NH_4_NO_3_–free MS medium (M0238, Duchefa, RV Haarlem, The Netherlands), medium where NH_4_NO_3_ is fully substituted by NaNO_3_ (M0239, Duchefa, RV Haarlem, The Netherlands), and medium containing 10mM NH_4_+30mM NO_3_ (M0240, Duchefa, RV Haarlem, The Netherlands). Shoot tips in this experiment were cryoprotected and washed in S-35% solution but not cryopreserved (LNC only).

*Experiment 3. Impact of individual protocol steps and vitrification solution treatment.* In addition to a standard procedure, a set of treatments (Table 4) was designed to test the response of cryoprotected shoot tips to modification or omission of individual protocol steps and various vitrification solution treatments combined with regrowth medium with or without ammonium ion. In this experiment, both cryoprotected (LNC) and cryopreserved (LN) shoot tips were evaluated as indicated in Table 4. In the treatment for container modification, shoot tips were cryopreserved in 2-mL cryovials filled with vitrification solution, kept in LN for 1 h and rewarmed in a pre-heated (40 °C) water bath; shoot tips were withdrawn from the vials, washed in S-35% solution and recovered as described above in the standard procedure.

*Experiment 4. Effect of growth regulators in regrowth medium.* With the standard cryopreservation protocol, different combinations of growth regulators, GA_3_, BA, zeatin and indole acetic acid (IAA), were tested during the initial two steps of the shoot tip regrowth after cryoprotection and cryopreservation (Figure 1). The third regrowth step was performed using growth regulator-free medium as described in the standard procedure.

*Experiment 5. Effect of ammonium-free medium and growth regulators during three regrowth steps.* Based on the results of Experiment 4, effect of omission of ammonium nitrate at regrowth step 1 was tested on the background of presence or absence of growth regulators (1 mg/L GA_3_ + 1 mg/L BA) in regrowth medium used for three sequential regrowth steps (Table 3).

Compositions of cryoprotectant solutions used in the experiments are given in Table 5. All solutions were made on the basis of MS medium with or without NH_4_NO_3_ depending on the experiment (pH = 5.8) and filter-sterilized through Nalgene filters under vacuum (pore size 0.2–0.8 µm). All the chemicals were of analytical grade and purchased from Duchefa (The Netherlands). 

### 4.3. Recovery Assessment and Statistical Analysis

Survival was evaluated 2 weeks following cryoprotection (LNC) and cryopreservation (LN) by counting the number of shoot tips showing regrowth of green tissues. Regeneration was determined after 6 weeks, when the shoots had developed into normal plantlets (≥8 mm) with fully developed leaves and roots. Ten to 15 shoot tips were used per experimental condition and the experiments were replicated 3–5 times. 

Data from all experiments were analyzed by analysis of variance (ANOVA) and Duncan’s multiple range test (*p* < 0.05) using SAS on Demand for Academics software (SAS Institute Inc., Cary, NC, USA). Results are presented as percentages with their standard deviations.

## 5. Conclusions

A systematic approach presented in this study enables fast optimization of a complicated multi-stage cryopreservation protocol for plants with a limited number of starting materials, such as *P. yatabeanus*, an endangered Korean species. The highest shoot tip survival (92%) and plant regeneration (90%) after cryopreservation were achieved using preculture with 10% sucrose followed by 40 min osmoprotection and 60 min treatment with vitrification solution A3-80% on ice. Development of healthy plantlets from cryopreserved shoot tips required a three-step regrowth procedure starting with ammonium-free medium with GA_3_ and BA, followed by ammonium-containing medium with and without growth regulators. The presence of ammonium nitrate and its full or partial substitution by NaNO_3_ during the initial regrowth step inhibited regeneration of both cryoprotected and cryopreserved shoot tips, while the presence of ammonium during preculture and cryoprotection was necessary for normal regeneration. Among growth regulators tested, a combination of 1 mg/L GA_3_ and 1 mg/L BA during regrowth steps 1 and 2 resulted in highest regeneration. 

Since all vitrification-based methods (droplet-vitrification, vitrification, encapsulation-vitrification, D- and V-cryoplate) share similar protocol steps, this approach may open the door for faster and more efficient optimization of the cryopreservation procedures for new plant species with limited availability of starting material. This approach can also be used as a preliminary study to assess the feasibility of cryopreservation technique for new species. We also propose the hypothesis that plant materials that are sensitive to osmotic and chemical toxicity of cryoprotectants may benefit from the step-wise regrowth starting with ammonium-free medium with growth regulators.

## Figures and Tables

**Table 1:** Effect of presence (+) or exclusion (−) of ammonium nitrate at various steps of the droplet-vitrification procedure and in the regrowth medium on survival and regeneration of *Pogostemon yatabeanus* shoot tips. Shoot tips were cryoprotected but not cryopreserved.

No.	Protocol Steps			Survival (%)	Regeneration (%)
	Preculture + Osmoprotection *	Cryoprotection + Unloading *	Regrowth (Step 1) **		
1	−	−	− (5 days)	91.3 ± 8.4 ^a^	70.1 ± 5.4 ^c^
2	−	−	+ (5 days)	87.5 ± 4.8 ^a^	71.6 ± 4.8 ^c^
3	+	−	− (5 days)	96.4 ± 3.1 ^a^	82.6 ± 2.9 ^b^
4	+	+	− (5 days)	97.7 ± 3.1 ^a^	95.7 ± 4.1 ^a^
5	+	+	− (3 days)	95.0 ± 2.7 ^a^	87.8 ± 7.5 ^ab^
6	+	+	− (1 day)	94.3 ± 5.4 ^a^	87.2 ± 7.9 ^ab^
7	+	+	+ (5 days)	97.1 ± 3.2 ^a^	63.3 ± 6.2 ^c^

* Murashige and Skoog (MS) medium with (+) or without (−) NH_4_NO_3_ was used as a base for preparing cryoprotectant solutions. ** Ammonium-free (−) or ammonium-containing (+) MS medium with growth regulators, 1 mg/L gibberellic acid (GA_3_) + 1 mg/L benzyl adenine (BA), applied during the first 1–5 days of the regrowth (step 1 in a three-step regrowth procedure).

**Table 2:** Effect of substitution of ammonium ion and different combinations of regrowth conditions on survival and regeneration of *Pogostemon yatabeanus* shoot tips in the droplet-vitrification procedure. Shoot tips were cryoprotected but not cryopreserved.

No.	Nitrogen Source in Cryoprotectant Medium *	Regrowth Media, Duration and Light/Dark Conditions **	Survival (%)	Regeneration (%)
1	NH_4_NO_3_	RM2, 5 d → RM2, 23 d → MSF, 14 d	90.6 ± 2.5 ^a^	70.0 ± 3.0 ^e^
2	NH_4_NO_3_	RM1, 5 d → RM2, 23 d → MSF, 14 d	97.5 ± 4.0 ^a^	96.2 ± 4.5 ^a^
3	NH_4_NO_3_	RM1, 5 d → RM2, 7 d → RM2, 7 d → RM2, 7 d → MSF, 14 d	93.3 ± 5.4 ^a^	94.6 ± 6.1 ^ab^
4	NH_4_NO_3_	NaNO_3_ in RM1, 5 d → RM2, 23 d → MSF, 14 d	90.4 ± 6.1 ^a^	80.1 ± 5.6 ^cd^
5	NaNO_3_	NaNO_3_ in RM1, 5 d → RM2, 23 d → MSF, 14 d	93.4 ± 4.1 ^a^	87.7 ± 4.8 ^abc^
6	NH_4_NO_3_	(10 mM NH_4_+30 mM NO_3_) in RM, 5 d → RM2, 23 d→ MSF, 14 d	93.2 ± 7.3 ^a^	80.5 ± 1.6 ^cd^
7	NH_4_NO_3_	RM2+AC, 5 d → RM2, 23 d → MSF, 14 d	89.2 ± 3.7 ^a^	73.3 ± 9.0 ^de^
8	NH_4_NO_3_	RM1+AC, 5 d → RM2, 23 d → MSF, 14 d	92.5 ± 1.4 ^a^	87.7 ± 4.2 ^abc^
9	NH_4_NO_3_	RM2, L1, 5 d → RM2, 23 d → MSF, 14 d	90.2 ± 2.8 ^a^	75.6 ± 6.9 ^de^
10	NH_4_NO_3_	RM1, L1, 5 d → RM2, 23 d→ MSF, 14 d	92.9 ± 4.0 ^a^	85.8 ± 4.2 ^bc^

* Sucrose and cryoprotectant solutions used at preculture, osmoprotection, cryoprotection and unloading stages of the cryopreservation protocol were prepared based on MS medium with NH_4_NO_3_ or NaNO_3_ as a nitrogen source. ** RM1, NH_4_NO_3_–free MS medium with growth regulators (1 mg/L GA_3_ + 1 mg/L BA); RM2, ammonium-containing MS medium with growth regulators (1 mg/L GA_3_ + 1 mg/L BA); MSF, ammonium-containing MS medium without growth regulators. AC, activated charcoal 1.5 g/L; L1, light provided by one fluorescent lamp, 40 µE/(m^2^ s); d—days

**Table 3:** Effect of ammonium nitrate and growth regulators at three regrowth steps on survival and regeneration of cryoprotected (LNC) and cryopreserved (LN) *Pogostemon yatabeanus* shoot tips.

No.	Regrowth Steps *				LNC		LN	
	Step 1 5 Days		Step 2 23 Days	Step 3 14 Days	Survival (%)	Regeneration (%)	Survival (%)	Regeneration (%)
	NH_4_NO_3_	GA_3_ + BA	GA_3_ + BA	GA_3_ + BA				
1	−	+	+	−	95.0 ± 7.1 ^a^	100.0 ± 0.0 ^a^	94.9 ± 6.8 ^a^	91.6 ± 1.0 ^a^
2	−	+	−	−	95.0 ± 7.1 ^a^	45.0 ± 7.1 ^d^	81.9 ± 10.3 ^ab^	32.2 ± 1.9 ^d^
3	−	−	+	−	90.0 ± 0.0 ^a^	75.0 ± 7.1 ^c^	77.6 ± 7.3 ^ab^	56.7 ± 17.9 ^bc^
4	−	−	−	−	95.0 ± 7.1 ^a^	88.8 ± 1.8 ^ab^	71.2 ± 5.2 ^bc^	35.4 ± 6.5 ^cd^
5	+	+	+	−	95.0 ± 7.1 ^a^	53.6 ± 5.1 ^d^	59.5 ± 18.1 ^cd^	50.0 ± 14.1 ^bcd^
6	+	−	−	−	77.9 ± 11.1 ^a^	53.6 ± 5.1 ^d^	53.0 ± 13.0 ^d^	7.9 ± 2.9 ^e^
7	−	+	+	+	95.0 ± 7.1 ^a^	85.0 ± 7.1 ^bc^	93.4 ± 5.9 ^a^	69.8 ± 7.6 ^b^

* Step 1 was performed on MS medium with (+) or without (−) growth regulators (1 mg/L GA_3_ +1 mg/L BA) and/or ammonium nitrate in darkness. Steps 2 and 3 were performed on MS medium containing ammonium nitrate with (+) or without (−) 1 mg/L GA_3_ + 1 mg/L BA under light, 40 µE/(m^2^ s).

**Table 4:** Set of treatments in Experiment 3 to test the impact of individual stages of the droplet-vitrification protocol and vitrification solution treatments in combination with regrowth medium with (RM2) or without (RM1) ammonium nitrate for cryopreservation of *Pogostemon yatabeanus* shoot tips. Except for treatments listed in the table, other steps of the cryopreservation protocol remained the same as described for standard procedure in Materials and Methods. LNC indicated shoot tips that were cryoprotected through the droplet-vitrification protocol but not cryopreserved. LN includes exposure to liquid nitrogen and rewarming.

Pre-LN Step	Treatments *	Regrowth Medium **	Code	LNC	LN
Preculture	No preculture	RM1	noPC	+	+
		RM2		+	
	10% sucrose 31 h → 25% sucrose 17 h	RM1	S-25%	+	+
		RM2		+	
	10% sucrose 31 h	RM1	standard	+	+
		RM2		+	
	10% sucrose 31 h → 17.5% sucrose 17 h	RM1	S-17.5%		+
Osmoprotection	No osmoprotection	RM1	noOP	+	+
		RM2		+	
	C4-35% 40 min	RM1	standard	+	+
Cryoprotection	No vitrification solution treatment	RM1	noVS	+	+
		RM2		+	
	A3-80% ice 60 min	RM1	A3-80% ice 60 standard	+	+
		RM2		+	
	A3-80% ice 30 min	RM1	A3-80% ice 30	+	
		RM2		+	
	A3-80% ice 90 min	RM1	A3-80% ice 90	+	+
		RM2		+	
	A3-80% RT 20 min	RM1	A3-80% RT 20	+	
		RM2		+	
	B5-80% RT 60 min	RM1	B5-80% RT 60	+	+
		RM2		+	
	B1-100% RT 60 min	RM1	B1-100% RT 60	+	
		RM2		+	
Container	Cryovial, 2 mL	RM1	Vial	−	+

* Composition of cryoprotectant solutions is given in Table 5; RT—room temperature, 25 ± 1 °C; ice—treatment performed on the ice bath at 0 °C. ** Regrowth medium used during the first 5 days after rewarming (step 1 of the three-step standard regrowth procedure). RM1, NH_4_NO_3_–free MS medium with growth regulators (1 mg/L GA_3_ + 1 mg/L BA); RM2, ammonium-containing MS medium with growth regulators (1 mg/L GA_3_ + 1 mg/L BA).

**Table 5:** Composition of solutions used for preculture, osmoprotection, cryoprotection and unloading.

Solution	Composition (%, *w*/*v*) *	Total Concentration of Cryoprotectants (%, *w*/*v*)
S-10%	S 10.0	10.0
S-35%	S 35.0	35.0
C4-35%	G 17.5 + S 17.5	35.0
A1-73.7% (PVS2)	G 30.0 + DMSO 15.0 + EG 15.0 + S 13.7	73.7
A3-90%	G 37.5 + DMSO 15.0 + EG 15.0 + S 22.5	90.0
A3-80%	G 33.3 + DMSO 13.3 + EG 13.3 + S 20.1	80.0
A3-70%	G 29.2 + DMSO 11.7 + EG 11.7 + S 17.4	70.0
B1-100% (PVS3)	G 50.0 + S 50.0	100.0
B5-80%	G 40.0 + S 40.0	80.0

* G, glycerol; S, sucrose; DMSO, dimethyl sulfoxide; EG, ethylene glycol. All solutions were prepared on the basis of MS medium, pH was adjusted to 5.8 before filter sterilization.

## Data Availability

Not applicable, no new data sets were generated during the study.

## Supplementary Materials

The following are available online at https://www.mdpi.com/article/10.3390/plants10102018/s1, Figure S1: (Complementary to Table 3). Effect of ammonium nitrate and growth regulators at three regrowth steps on plant regeneration of cryoprotected (LNC) and cryopreserved (LN) *Pogostemon yatabeanus* shoot tips. Photographs were taken 6 weeks after treatment. Data on survival and regeneration are presented in Table 3.
