# Supplementary material for: Optimization of a Cryopreservation Method for the Endangered Korean Species Pogostemon yatabeanus Using a Systematic Approach: The Key Role of Ammonium and Growth Regulators
Source: Plants (Basel). 2021 Sep 26;10(10):2018. doi: 10.3390/plants10102018 (PMC8538529; doi:10.3390/plants10102018)
Supplement: Supplementary file 1 [file plants-10-02018-s001.zip › plants-1388900-supplementary.pdf]

Supplementary materials

Figure S1 (Complementary to Table 3). Effect of ammonium nitrate and growth regulators at three regrowth steps on plant regeneration of cryoprotected (LNC) and cryopreserved (LN) *Pogostemon yatabeanus* shoot tips. Photographs were taken 6 weeks after treatment. Data on survival and regeneration are presented in Table 3.

No.	Regrowth steps*				LNC	LN
	Step 1 5 days		Step 2 23 days	Step 3 14 days		
	NH_4NO_3	GA_3+BA	GA_3+BA	GA_3+BA		
1	-	+	+	-	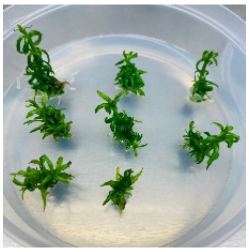	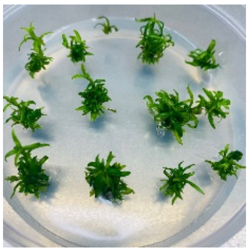
2	-	+	-	-	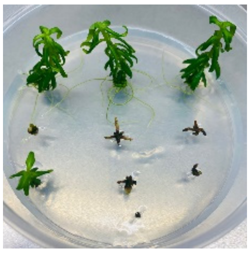	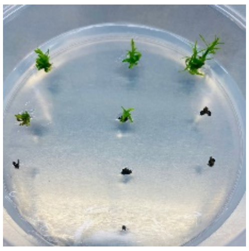
3	-	-	+	-	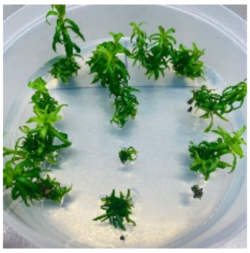	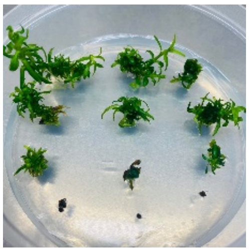
4	-	-	-	-	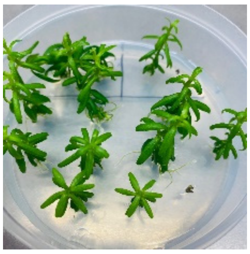	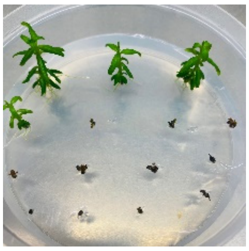
5	+	+	+	-	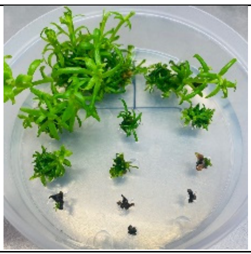	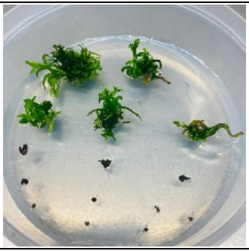

6	+	-	-	-	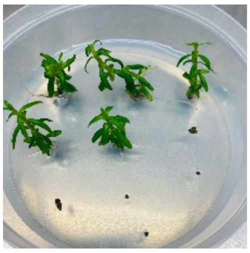	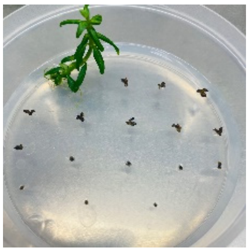
7	-	+	+	+	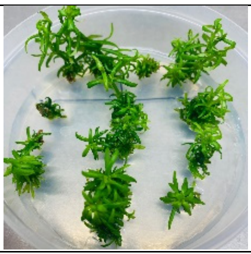	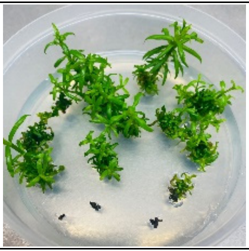

* Step 1 was performed on MS medium with (+) or without (-) growth regulators (1 mg/l GA_3 +1 mg/l BA) and/or ammonium nitrate in darkness. Steps 2 and 3 were performed on MS medium containing ammonium nitrate with (+) or without (-) 1 mg/l GA_3 + 1 mg/l BA under light (40 µE m^{-2} s^{-1}).
